# Addressing the drug-resistant tuberculosis challenge through implementing a mixed model of care in Uganda

**DOI:** 10.1371/journal.pone.0244451

**Published:** 2020-12-29

**Authors:** Samuel Kasozi, Nicholas Sebuliba Kirirabwa, Derrick Kimuli, Henry Luwaga, Enock Kizito, Stavia Turyahabwe, Deus Lukoye, Raymond Byaruhanga, Lisa Chen, Pedro Suarez

**Affiliations:** 1 TRACK TB Project, Management Sciences for Health, Kampala, Uganda; 2 National Tuberculosis and Leprosy Program, Ministry of Health, Kampala, Uganda; 3 Curry International Tuberculosis Center (UCSF/CITC), University of California, San Francisco, San Francisco, California, United States of America; 4 Management Sciences for Health, Arlington, Virginia, United States of America; Georgia Southern University, UNITED STATES

## Abstract

Worldwide, Drug-resistant Tuberculosis (DR-TB) remains a big problem; the diagnostic capacity has superseded the clinical management capacity thereby causing ethical challenges. In Sub-Saharan Africa, treatment is either inadequate or lacking and some diagnosed patients are on treatment waiting lists. In Uganda, various health system challenges impeded scale-up of DR-TB care in 2012; only three treatment initiation facilities existed, with only 41 of the estimated 1010 RR-TB/MDR-TB cases enrolled on treatment yet 300 were on the waiting list and there was no DR-TB treatment scale-up plan. To scale up care, the National TB and leprosy Program (NTLP) with partners rolled out a DR-TB mixed model of care. In this paper, we share achievements and outcomes resulting from the implementation of this mixed Model of DR-TB care. Routine NTLP DR-TB program data on treatment initiation site, number of patients enrolled, their demographic characteristics, patient category, disease classification (based on disease site and human immunodeficiency virus (HIV) status), on co-trimoxazole preventive therapy (CPT) and antiretroviral therapy (ART) statuses, culture results, smear results and treatment outcomes (6, 12, and 24 months) from 2012 to 2017 RR-TB/MDR-TB cohorts were collected from all the 15 DR-TB treatment initiation sites and descriptive analysis was done using STATA version 14.2. We presented outcomes as the number of patient backlog cleared, DR-TB initiation sites, RR-TB/DR-TB cumulative patients enrolled, percentage of co-infected patients on the six, twelve interim and 24 months treatment outcomes as per the Uganda NTLP 2016 Programmatic Management of drug-resistant Tuberculosis (PMDT) guidelines (NTLP, 2016). Over the period 2013–2015, the RR-TB/MDR-TB Treatment success rate (TSR) was sustained between 70.1% and 74.1%, a performance that is well above the global TSR average rate of 50%. Additionally, the cure rate increased from 48.8% to 66.8% (P = 0.03). The Uganda DR-TB mixed model of care coupled with early application of continuous improvement approaches, enhanced cohort reviews and use of multi-disciplinary teams allowed for rapid DR-TB program expansion, rapid clearance of patient backlog, attainment of high cumulative enrollment and high treatment success rates. Sustainability of these achievements is needed to further reduce the DR-TB burden in the country. We highly recommend this mixed model of care in settings with similar challenges.

## Introduction

Worldwide, Drug-resistant Tuberculosis (DR-TB) remains a big challenge with Multidrug-resistant TB (MDR-TB) and extensively drug-resistant TB (XDR-TB) being the worst forms. DR-TB is said to occur when TB organisms can continue to grow in the presence of one or more anti-TB drugs. Different forms of DR-TB exist and these include; Mono resistance, Poly resistance, Rifampicin resistance (RR-TB), MDR-TB and XDR-TB. Mono-resistance: resistance to one first-line anti-TB drug only while Poly-resistance: resistance to more than one first-line anti-TB drug, other than both isoniazid and rifampicin. MDR-TB is defined as TB that is resistant to at least the two most powerful first-line medicines (Rifampicin and Isoniazid) while XDR-TB is a form of TB that addition to being resistant to Rifampicin and Isoniazid, is resistant to any Fluoro-quinolone and at least one of the injectable second-line drugs. Rifampicin resistance (RR-TB): resistance to rifampicin detected using phenotypic or genotypic methods, with or without resistance to other anti-TB drugs; includes any resistance to rifampicin in the form of mono-resistance, poly-resistance, MDR-TB, or XDR-TB [1–3]. In 2015 alone, about 580,000 people developed MDR-TB and about 9.7% of these cases had XDR-TB [4] while an estimated 250,000 people died of MDR-TB [1, 4]. Notable causes of DR-TB among others include; poor adherence to TB treatment, inappropriate or incorrect use of anti-TB drugs and the use of poor quality medicines [5]. Again, due to the growing availability of rapid diagnostics (Xpert MTB/Rif assay), the detection and diagnosis of DR-TB patients is on the increase. However, in most settings, the diagnostic capacity has superseded the DR-TB clinical management capacity thereby causing ethical challenges [6, 7]. Untreated MDR-TB fuels the generation and subsequent transmission of XDR-TB [2] and incident cases are predicted to increase [8]. In this regard, the emergence of DR-TB continues to threaten global efforts to eliminate TB and threatens to reverse the global progress made in TB control [9–12].

In Sub-Saharan Africa, a resource-limited setting, the true burden of DR-TB is unknown as most countries have not conducted drug-resistance surveys. To make matters worse, the treatment of patients with DR-TB is either inadequate or lacking [2, 13]. This precedent has resulted in DR-TB patients diagnosed being put on treatment waiting lists as affected countries try to establish or scale up PMDT treatment programs. The outcome of this is that most DR-TB patients are delayed to start treatment resulting in high morbidity and mortality [14–17].

Uganda is one of the “30 high burden TB/ Human immunodeficiency virus (HIV)” countries that collectively account for 90% of the global TB burden [18]. While the World Health Organization (WHO) had removed Uganda off the list of TB high burden countries (HBC) in 2015 [20], a recent Uganda population-based TB prevalence survey suggests that incidence and prevalence rates in the country are far higher than previously believed [19, 20], and that notification rates for drug-susceptible (DS) and DR-TB represent only a small proportion of actual cases [20]. In 2012, the Uganda National TB and Leprosy Programme (NTLP) had encountered several challenges in implementing TB control activities in the country, for example, there was limited capacity to rapidly clear the 300 RR-TB/MDR-TB patient backlogs. Consequently, in 2013, the NTLP was supported to overcome such challenges [20, 21]. Partners provided both logistical and technical support to NTLP central unit, treatment sites and played a coordination role. Therefore, the objective of this descriptive review was to assess and share resulting from the achievements of the implementation of the Uganda mixed Model of DR-TB care.

## Materials and methods

### Study area

Uganda is a landlocked country, located in East Africa with a total population of 34.6 million and over 111 districts and one City [22]. Uganda is among the 30 high TB/HIV burden countries [18] with a TB prevalence and incidence at 253 cases per 100,000 and 234 cases per 100,000 respectively [19, 23]. The burden of RR-TB/MDR-TB is estimated at 1.4% among all new and 12.1% among previously treated TB cases [9, 11]. In 2012, Uganda notified about 310 out of an estimated 1010 RR-TB/MDR-TB cases. Of these, only 41 were enrolled on treatment, and about 300 RR-TB/MDR-TB patients awaited treatment during this period [17, 21]. The lack of treatment for most RR-TB/MDR-TB patients perpetuated an ongoing transmission posing a major threat to the community around these patients. During the same period, the NTLP was still grappling with a lack of a nationally agreed DR-TB scale-up plan coupled with a weak health care system. There was limited access to drug susceptibility test (DST), unreliable second-line drug (SLD) management system, with no contact tracing of contacts of index cases besides limited expertise in case management and poor access to treatment. More still, the country only had three DR-TB treatment initiation facilities [21, 24] and thus health workers often referred RR-TB/MDR-TB patients to these few treatment initiation sites. Furthermore, there were low levels of sputum follow up examinations; loss of specimens during transportation; drug stock-outs; lack of appropriate isolation spaces, and limited funding at both NTLP central unit and district levels. Due to high stigma towards RR-TB/MDR‐TB patients and high mobility of populations, RR-TB/MDR‐TB patient loss‐to‐follow‐up was high [21].

### Study design

#### Service delivery through a mixed DR-TB model of care

The above challenges and the need to expand the Programmatic Management of DR-TB (PMDT) program led to a paradigm shift. The NTLP and partners (USAID funded TRACK TB Project, Strengthening Uganda's Systems for Treating AIDS Nationally/SUSTAIN and The Global Fund to Fight AIDS, Tuberculosis, and Malaria/GFATM) designed and scaled up DR‐TB service delivery [21]. A locally appropriate Uganda-specific mixed model of DR-TB treatment was designed and rolled by NTLP and partners to rapidly absorb the RR-TB/MDR-TB patient backlog to save lives and to curtail the ongoing DR-TB transmission [25]. This DR-TB mixed model of care involves brief periods of hospitalization (in-patient) followed a long period of ambulatory/clinic-based care. Patients who are severely ill or not within the immediate catchment area of the treatment initiation hospital are admitted for a short period of 1–8 weeks and thereafter are transferred for ambulatory care at a prepared peripheral directly observed therapy (DOT) follow up facility nearest to patients’ homes. In this paper, we share experiences, implementation approach, achievements, and lessons/good practices from the implementation of the mixed DR-TB treatment model of care in Uganda starting from 2013 through 2017. This model may be ideal for both current and future PMDT programs in similar resource settings.

#### Interventions, innovations, and roll out of the DR-TB mixed model

Before the DR-TB program was scaled up, a baseline analysis was conducted that informed the DR-TB minimum package of interventions that were applied. These interventions included the strengthening of health care provider skills, implementation of quality improvement and use of standard operating procedures, improving access to patient investigation/treatment monitoring tests, management of TB commodities, strengthening of data management, the performance of enhanced cohort reviews, improving TB Infection Control practices, facilitation to implement ambulatory care (Home visiting, contact tracing, follow up facility training/mentorship and drug delivery), general hospital administrative support and provision of patient psychosocial economic support including food and transport (incentives and enablers). This direct patient socio-economic support is reported to be associated with better treatment outcomes through enhancement of nutritional status, patient adherence, and compliance [26, 27].

NTLP was supported with logistical and technical support at both its central unit and at all DR-TB facilities. Human resource (HR) capacity and partner coordination at NTLP central unit was strengthened through the secondment of technical staff to lead NTLP continuous quality improvement (CQI) campaigns and overall partner coordination. Early implementation of continuous quality improvement campaigns through peer to peer mentorships, coaching, holding learning sessions contributed to improved quality of care and the building of multi-disciplinary teams of DR-TB experts in sites where few experts existed. Therefore, these teams of experts provided the day to day advisory services at both the national and regional levels. More still, initially, a toll-free helpline was established to support the linkage of peripheral regional site teams to the National DR-TB Panel for specialized advisory services until an Echo platform which a learning network, established by the Project ECHO (University of New Mexico) was introduced.

To ensure standardized DR-TB program implementation, DR-TB treatment guidelines, and other tools were developed and disseminated through National coordination committee (NCC) meetings at the central level while at the subnational level, this was achieved through performance and cohort review meetings. Conduction of enhanced cohort reviews also helped to validate program data, identify performance gaps and best practices. Therefore, these enhanced cohort reviews served as a vehicle for capacity building and programmatic implementation of continuous quality improvement (CQI) campaigns.

More human resource capacity at DR-TB treatment initiation was built based on a standardized curriculum and training materials including at Follow up health facilities (FUFs, defined as any health facility identified and accepted to offer DOT to DR-TB patients). Remodelling/construction of DR-TB wards to both expand admission ward space as well as improve TB infection control was supported. Community linkage facilitators (CLF) and district teams supported adherence enhancement through the provision of patient and family education on DR-TB disease, tracking of patients lost to follow-up (LTFU) as well as linking of diagnosed patients. The DR-TB program monitoring and evaluation system was strengthened through the development of an electronic web-based recording/reporting management system (DR-TB Management Information System). To strengthen the DR-TB drug and commodity supply chain, the QUAN TB tool was used [31] to improve ordering as well as monitoring of utilization of DR-TB commodities. Again, the installation of GeneXpert machines while ensuring internet connectivity to improve access to rapid DST (drug susceptibility testing) and to facilitate the real-time transmission of GeneXpert results through the GxAlert information system was done. To ensure a high DR-TB program quality, NTLP’s central DR-TB core committee that is part of the national technical committee that oversees implementation, policy formulation, resource mobilization, implementation science and overall coordination of the PMDT program was set up and facilitated to hold regular coordination meetings during which action plans were drawn. For Treatment Initiation Sites (TISs) and, FuFs’ roles see Table 1.

**Table 1:** Roles of treatment initiation sites and FuFs in DR-TB management.

Treatment initiation site (TIS)	Follow up Facility (FuF)
• Initiating and offering “start-to-finish” case management (monthly clinic reviews, monitoring and managing side effects, offering DR-TB expert Panel services, and conducting cohort reviews to assign treatment outcomes)	• Providing DOT to DR-TB patients on ambulatory care
	• Providing adherence counselling and education to patients and families
	• Conducting patients’ home visits for contact tracing, nutrition, and infection control assessments.
• Training and mentoring FuFs for ambulatory care	
• Managing and supplying DR-TB commodities to all initiated/active patients	

DR-TB, Drug-resistant tuberculosis.

### Data management and analysis

We used the baseline program data collected in 2012 and the routine DR-TB program 2013–2017 data collected by NTLP from all the 15 sites to describe the achievements and outcomes of implementing the DR-TB mixed model in Uganda. Data on the following variables were collected; treatment initiation site, RR-TB/MDR-TB patients enrolled, sex, age, patient categories, disease type, HIV status, HIV integration, culture and smear results, 6-month interim outcomes, 12-month interim outcomes and 24-month final treatment outcomes based on Uganda NTLP guidelines [32]. The study endpoints for the six, twelve and 24-month outcome analysis were 12, 18 and 24 months after the closing day of the RR-TB/MDR-TB cohort enrollment respectively. Based on these study endpoints, RR-TB/MDR-TB cohort data from 2012 to 2017 for all the 15 treatment initiation facilities were collected and descriptive analysis was done using STATA version 14.2 (Stata Corp. 2015. Stata Statistical Software: College Station, TX: Stata Corp LP). Continuous data were summarized using medians while categorical data were computed as proportions. We presented the data in text, tables, and graphs. Results for trends were presented at p-value < 0.05 level of significance and 95% confidence interval. We considered the achievements and outcomes to be; the increase in the number of RR-TB/MDR-TB patient backlog cleared, an increase in the number of DR-TB initiation sites, an increase in cumulative number patients enrolled, an increasing percentage of HIV integration interventions and favourable six, twelve months interim and 24 months final treatment outcomes among 2013–2017 cohorts. The RR-TB/MDR-TB outcome measures were defined according to the Uganda NTLP 2016 PMDT guidelines [32].

### Ethical considerations

We used routine NTLP program data [19, 23, 24] collected for routine patient care at all health facilities in Uganda with no patient interaction. The data did not carry any personal identifiers and third parties had no access to this data. Permission to use the data for this study was sought from the NTLP. The researchers did not anticipate any risk or benefit to the patients in the analysis of this information.

## Results

**Fig 1:**
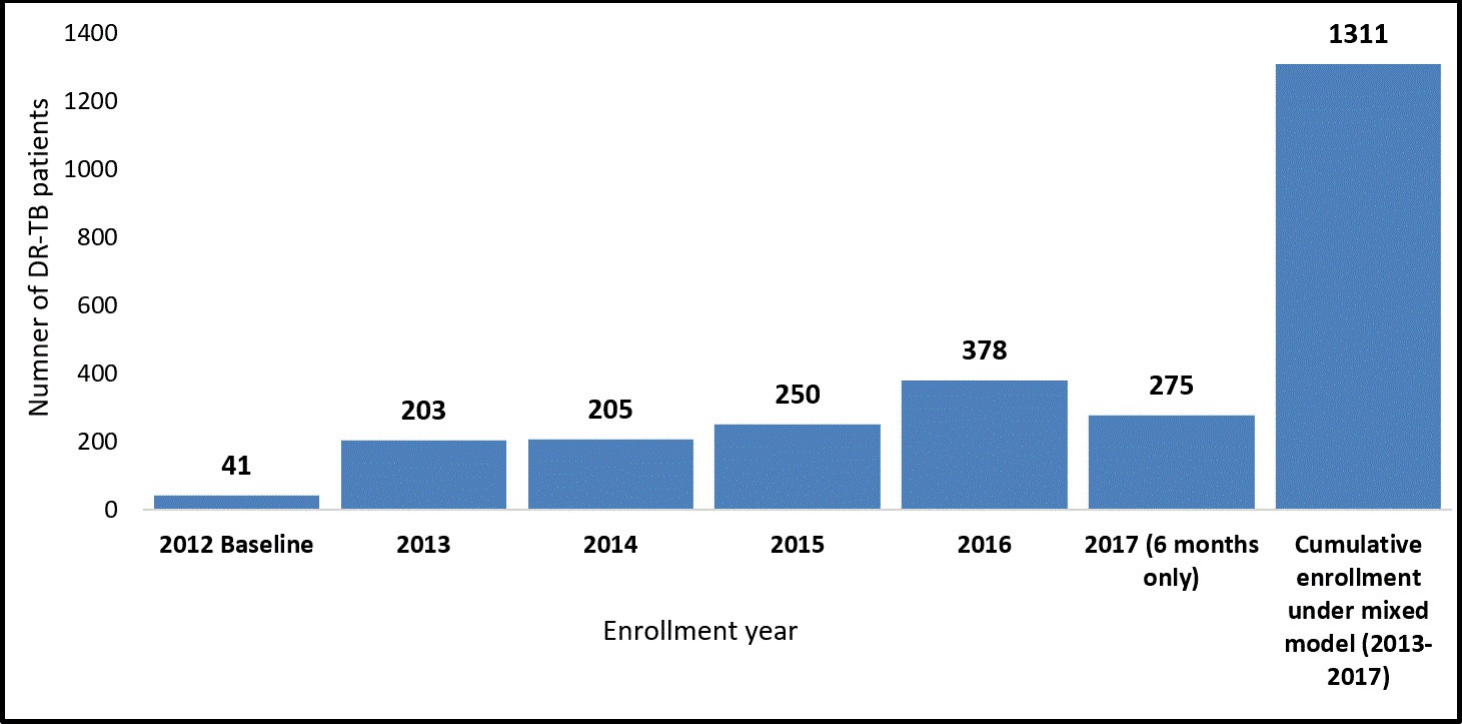
The number of RR-TB/MDR-TB patients enrolled by year.

**Fig 2:**
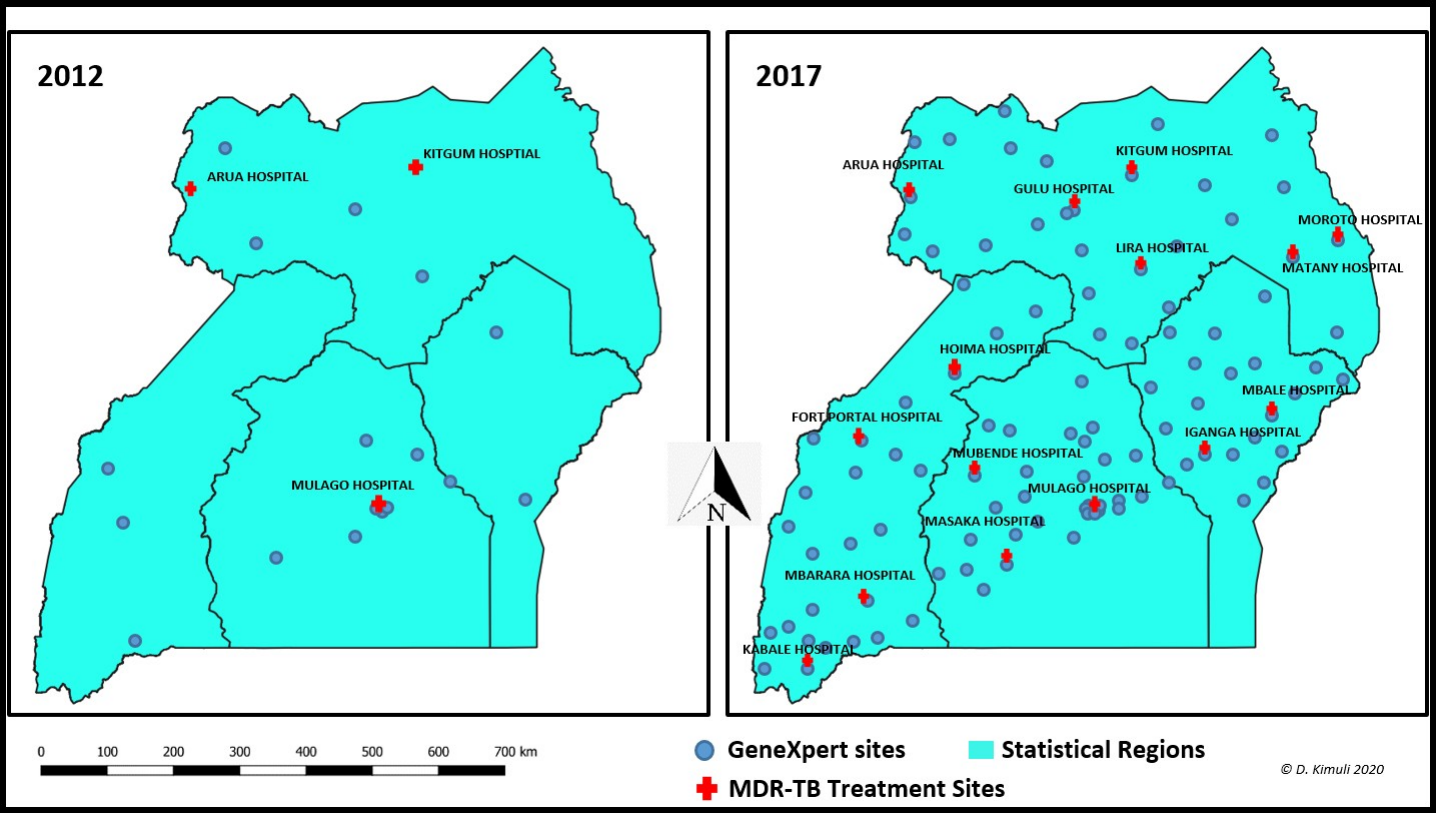
GeneXpert machines and DR-TB treatment sites in 2012 and 2017.

The backlog of the 300 RR-TB/MDR-TB patients who were on the treatment waiting list was cleared by the end of 2014. The RR-TB/MDR-TB cumulative patient enrollment increased from 41 in 2012 to 1311 patients (Fig 1) in 2017. GeneXpert coverage increased from 24 GeneXpert machines in 2012 to 131 GeneXpert machines in 2017 (Fig 2). The operationalization of the Uganda DR-TB mixed model also allowed for the rapid expansion of the DR-TB treatment and care program from 3 DR-TB treatment initiation sites at baseline in 2012 to 12 sites in 2013, 15 sites in 2017 (Fig 2).

### Characteristics of patients enrolled for RR-TB/MDR-TB treatment

The characteristics of the RR-TB/MDR-TB patients enrolled for treatment from 2013 to 2017 are shown in Table 2 below. In this period, a total of 1,311 RR-TB/MDR-TB patients were enrolled; 64.4% (844/1311) were male. The majority of enrolled patients, 47.2% (619/1311), were in the young and productive age group of 15–34 years. 41.7% (547/1311) were aged 35–54 years, 7.6% (100/1311) were older than 54 years and 3.3% (43/1311) were under 15 years. The median age was 34 years, with a mean of 35.53 ± SD 12.78 years. Most patients, 98.5% (1291/1311) had pulmonary RR-TB/MDR-TB, and slightly more than half of the patients enrolled from 2013 through 2017 52.6% (690/1311) were HIV co-infected.

**Table 2:** Characteristics of RR-TB/MDR-TB patients enrolled for treatment in 2012 and 2017.

Baseline characteristics	*2012 Baseline*	*2013*	*2014*	*2015*	*2016*	*2017*
	*n (%)*	*n (%)*	*n (%)*	*n (%)*	*n (%)*	*n (%)*
Total (N)	41	203	205	250	378	275
**Sex**						
Male	26(63.4)	126(62.1)	131(63.9)	168(67.2)	247(65.3)	172(62.6)
Female	15(36.6)	77(37.9)	74(36.1)	82(32.8)	131(34.7)	103(37.4)
**Age (years)**						
<15	2(4.9)	5(2.5)	3(1.5)	7(2.8)	12(3.2)	16(5.8)
15–34	13(31.7)	97(47.8)	88 (42.9)	132(52.8)	180(47.6)	122(44.4)
35–54	24(58.5)	91(44.8)	101(49.3)	96(38.4)	153(40.5)	106(38.5)
>54	2(4.9)	10(4.9)	13(6.3)	14(5.6)	33(8.7)	30(10.9)
Missing age	0(0.0)	0(0.0)	0(0.0)	1(0.4)	0(0.0)	1(0.4)
Pulmonary	39(95.1)	200(98.5)	201(98.1)	248(99.2)	373(98.7)	269(97.8)
Extra-pulmonary	2(4.9)	3(1.5)	4 (1.9)	2(0.8)	5(1.3)	6(2.2)
**HIV status**						
HIV-negative	28(68.3)	112(55.2)	96(46.8)	107(42.8)	167(44.2)	139(50.5)
HIV-positive	13(31.7)	91(44.8)	109(53.2)	143(57.2)	211(55.8)	136(49.5)

N/n = Number of patients.

HIV—human immunodeficiency virus.

### Treatment outcomes

#### Six-month interim treatment outcomes for RR-TB/MDR-TB patients enrolled from 2012 to 2016

Although the culture conversion rates initially improved at month six from 48.8% (20/41) in 2012 to 55.2% (112/203) in 2013 and 67.8% (139/205) in 2014, there was a drop off to 58.0% (145/250) in 2015 and 47.6% (180/378) in 2016 cohorts (Table 3).

**Table 3:** Six-month interim outcomes for RR-TB/MDR-TB patients enrolled from 2012 to 2016.

Interim outcomes at 6 months	*2012 Baseline*	*2013*	*2014*	*2015*	*2016*	*Total 2013–2016*
	*n (%)*	*n (%)*	*n (%)*	*n (%)*	*n (%)*	*n (%)*
Total	41	203	205	250	378	1036
Culture negative	20(48.8)	112(55.2)	139(67.8)	145(58.0)	180(47.6)	576(55.6)
Culture positive	1(2.4)	2(1.0)	1(0.5)	4(1.6)	2(0.5)	9(0.9)
Culture unknown	20(48.8)	89(43.8)	65(31.7)	101(40.4)	196(51.9)	451(43.5)

N/n = Number of patients.

#### Twelve-month interim outcomes for RR-TB/MDR-TB patients enrolled in 2012–2015

The culture conversion rates at 12 months initially improved from 29.3% (12/41) to 51.7% (105/203) in 2013 and 63.9% (131/205) in 2014 but again dropped to 48% (120/250) in 2015 (Table 4).

**Table 4:** Twelve-month interim outcomes for RR-TB/MDR-TB patients enrolled in 2012–2015.

Interim outcomes at12 months	*2012 Baseline*	*2013*	*2014*	*2015*	*Total 2013–2015*
	*n (%)*	*n (%)*	*n (%)*	*n (%)*	*n (%)*
Total	41	203	205	250	658
Culture negative	12(29.3)	105(51.7)	131(63.9)	120(48.0)	356(54.1)
Culture positive	0(0.0)	2(1.0)	1(0.5)	0(0.0)	3(0.5)
Culture unknown	29(70.7)	96(47.3	73(35.6)	130(52.0)	299(45.4)

N/n = Number of patients.

#### Final treatment outcomes for RR-TB/MDR-TB patients enrolled in 2012 and 2014

The original small cohort of 41 RR-TB/MDR-TB patients in 2012 cared for by partner organizations achieved a high level of treatment success of 78% (32/41), but the notable achievement under this NTLP led DR-TB mixed model is that NTLP managed to maintain high treatment success rates of 74.0% among both 2013 and 2014 cohorts and then 70.1% in the 2015 cohort in the face of the large rise in enrollment and rapid expansion to multiple sites across the country, within the public health system (Table 5). Evidence from other settings shows similar outcomes when patients are hospitalized for a brief period followed with ambulatory care so long as patient support and monitoring is done [28].

**Table 5:** Final treatment outcomes for RR-TB/MDR-TB patients enrolled in 2012 and 2015.

Final Treatment outcome	2012 Baseline	2013	2014	2015	P-value
	*n (%)*	*n (%)*	*n (%)*	*n (%)*	*(*X^2^*)*
Total	41	203	205	241	
Cured^¥^	20(48.8)	122(60.1)	142(69.3)	161(66.8)	0.03
Completed^Ø^	12(29.3)	28(13.8)	10(4.9)	8(3.3)	<0.05
TSR*	32(78.0)	150(73.9)	152(74.1)	169(70.1)	0.63
Unfavorable	9(22.0)	53(26.1)	53(25.9)	72(29.9)	
• Lost to Follow up	4(9.7)	15(7.4)	22(10.7)	34(14.1)	0.16
• Died	2(4.9)	36(17.7)	30(14.6)	34(14.1)	0.20
• Failed Treatment	0(0.0)	2(1.0)	1(0.5)	4(1.7)	0.57
• Not evaluated	3(7.3)	0(0.0)	0(0.0)	0(0.0)	<0.05
Tested for HIV	41(100)	203(100)	205(100)	241(100)	
HIV-positive	13(31.7)	91(44.8)	109(53.2)	138(57.3)	<0.05
Started on CPT	13(100)	91(100)	109(100)	138(100)	
Started on ART	13(100)	91(100)	109(100)	138(100)	

N/n = Number of patients. *From 2015 cohort 9 patients were found not RR/MDR patients and thus eliminated from final treatment outcomes

¥Cured is a pulmonary TB patient with bacteriologically confirmed TB at the beginning of treatment who was smear- or culture-negative in the last month of treatment and on at least one previous occasion.

Ø Treatment completed is a TB patient who completed treatment without evidence of failure BUT with no record to show that sputum smear or culture results in the last month of treatment and on at least one previous occasion were negative, either because tests were not done or because results are unavailable.

*TSR (Treatment Success Rate) is the sum of cure rate and completion rate.

HIV—human immunodeficiency virus, CPT—Co-trimoxazole preventive therapy, ART–antiretroviral therapy, *X*^2^ for the trend.

The proportion of RR-TB/MDR-TB patients that cured increased substantially from 48.8% in 2012 to 60% and above in 2013 to 2015 (*p* = 0.03), with a significant reduction in treatment completion rates from 29.3% to 3.3% (*p*<0.05) which points to improved quality of care. Overall, 1.1% (7/649) failed on treatment, with a slight increase in failure rate from 0% in 2012 to 1.7% in 2015 but this was not statistically significant (*p* = 0.57). Out of the 52.1% (338/649) HIV co-infected patients, 100% (338/338) were on both CPT and ART. However, HIV co-infection rates soared up from 31.7% in 2012 to 57.3% in 2015 (*p*<0.05), see Table 5.

## Discussion

The most notable achievement under this NTLP led DR-TB mixed model is that NTLP managed to improve the percentage of patients that were cured from 48.8% in 2012 to 66.8% in 2015. The NTLP also maintained high treatment success rates (TSR) of 74.0% among both 2013 and 2014 cohorts and 70.1% among the 2015 cohort. This rate was in comparison to the 78% TSR that was achieved among the original small cohort of 41 patients cared for by partner organizations under research setting in 2012. The achieved TSR was well above the global TSR average rate of 50% [4] and was achieved in the face of a high number of RR-TB/MDR-TB patient backlog (of 300 patients), large rise in enrollment and rapid expansion to multiple sites across the country within the public health system [24]. These high treatment success rates in 2013 through 2015 were possibly made possible through implementation of continuous quality improvement campaigns through peer to peer mentorships, coaching, holding learning sessions. These quality improvement campaigns contributed to improved quality of care and the building of multi-disciplinary teams of DR-TB experts. Besides, there was the conduction of enhanced cohort reviews which helped to validate program data, identify performance gaps and best practices. The quickly built DR-TB capacity through enhanced cohort reviews and early application of CQI approaches allowed for the rapid expansion of the DR-TB treatment initiation sites from three in 2012 to 12 in 2013 and subsequently to 15 from 2014 up to 2017. The significant rise in enrollment rates and in the proportion of cured RR-TB/MDR-TB patients over those who completed treatment attests to this built capacity and improved the quality of DR-TB care. Again, treatment models that embrace.

This rapid expansion in the number of DR-TB treatment initiation facilities and ambulatory care led to rapid clearance of the 300 RR-TB/MDR-TB patients who were on the waiting list since 2009 and the attainment of a cumulative RR-TB/MDR-TB enrollment of 1311 patients over five years (2013–2017) up from 41 enrolled in 2012. More still, widespread availability of GeneXpert machines enabled the identification of the 1311 RR-TB/MDR-TB cases despite a low GeneXpert utilization standing at an average of 6 tests per machine; there were 24 GeneXpert machines in 2012, 39 in 2013, 72 in 2014, 111 in 2015, 112 in 2016 and 131 in 2017. GeneXpert implementation is reported to improve early detection and to promote early treatment of RR-TB/MDR-TB [29–31].

According to Uganda PMDT guidelines, all newly diagnosed RR-TB/MDR-TB patients are started on a standard regimen - 6Km-Lfx-Cs-Eto-Z/14Lfx-Cs-Eto-Z until their DSTs are available at which point, their treatment becomes individualized based on observed resistance and side effects profiles [32]. Most patients responded well on this regimen as noticed through the improvement in the culture conversion rates between 2013 and 2015 compared to the 2012 baseline (*p*< 0.05). The majority of the patients had achieved culture conversion with only 0.9% (9/1036) still being culture positive. At 12 months, 54.1% (356/658) of the evaluated patients were culture-negative with only 0.5% (3/658) remaining culture positive. The high proportion of patients with no culture results at both six and 12 months outcome analysis across all the years is attributed to patient deaths, LTFU, and failures in sample referral system as well as failure to return culture and smear results to health facilities [21, 33]. Again, although decentralization of DR-TB treatment services was supposed to improve RR-TB/MDR-TB interim and final treatment outcomes, case holding was poorer in sites with big numbers of patient enrollment beyond site capacity. For instance, in 2016, an analysis of the performance at individual sites showed that sites which enrolled the highest number of patients had the highest number of deaths and patients Loss to follow up due to high volume of patients they have to manage [33]. Again, although these challenges were picked up by CQI assessments, addressing them could not be solved at once in a public health system.

Between 2013 and 2015, there were more patients that died while on treatment than were LTFU and failed treatment. Although one study attributed this death to TB-HIV co-infection [17], the high deaths and LTFU among the 2013 to 2015 cohorts could be attributed to; firstly, enrollment of critically ill patients as a result of being on a treatment waiting list for long before the expansion of PMDT in 2013 [20] and secondly, rapidly expanding PMDT program to DR-TB sites that had limited capacity and prior experience in managing RR-TB/MDR-TB patients in the bid to rapidly clear the patient backlog and save lives [20].

Of the 1,311 RR-TB/MDR-TB patients enrolled on treatment from 2013 up to 2017, 64.4% (844/1311) were males. This finding of a higher RR-TB/MDR-TB burden among males is also reported in other settings and the available epidemiological literature suggests that this differential burden may be due to environmental and biological factors [34–39]. However, in Uganda, whether this observation is due to environmental, biological, social determinants or increased male’s health-seeking behaviour remains to be fully established. Also, most of the patients were in the reproductive years, with pulmonary RR-TB/MDR-TB disease and were co-infected with HIV. This indicates a pressing need to identify and effectively manage RR-TB/MDR-TB patients with Pulmonary TB disease with a gender focus among men.

To reduce RR-TB/MDR-TB related mortality and DR-TB transmission, there is a need for early detection and prompt treatment of DR-TB patients. Again, the regularity in the provision of patient incentives and enablers under the Global Fund needs to be improved to lead to further improvement in RR-TB/MDR-TB treatment outcomes. Also, the increased emphasis on the provision of socio-economic support to improve treatment outcomes and overall quality of life is needed [12, 16, 40]. An improvement in the transportation of sputum samples to the central reference laboratory for testing, the turnaround time of results, and addressing logistical challenges is needed [20, 21, 23]. Therefore, more support should be extended to DR-TB sites to ensure that all data on baseline investigations, treatment monitoring including sputum smear and culture results and treatment outcomes are promptly and completely entered the DR-TB registers.

The strategies and approaches employed under this model are useful to most countries battling with the same problem of clearing backlog and expanding patient enrollment [25]. Available studies suggest that such DR-TB treatment models that embrace CQI approaches and decentralization of management of patients near or at home, are not only socially acceptable [27, 41] but are associated with favourable treatment outcomes; even among HIV co-infected and XDR-TB patients [42].

Due to the inherent nature of retrospective studies, this descriptive review had some limitations; some important study variables e.g. smear and culture monitoring results were unknown for a significant number of patients. Therefore, during study analysis, outcomes were assigned based on only records and monitoring data variables that were available in the DR-TB register. This has the potential of introducing a selection bias into our results, therefore, there is a need to strengthen data documentation especially now with the introduction of a web-based DR-TB management system. Finally, the absence of controls for such a review may lower the vigour for the conclusions drawn, however, this study may act as a benchmark for future studies.

## Conclusions

In conclusion, this descriptive review indicates that the NTLP led DR-TB mixed model of care achieved high TSRs of 74.0% among both 2013 and 2014 cohorts and 70.1% among 2015 cohort in the face of a high number of RR-TB/MDR-TB patient backlog (of 300 patients), large rise in enrollment and rapid expansion to multiple sites across the country within the public health system. These achievements are attributed to the early implementation of CQI campaigns which contributed to improved quality of care and the building of the capacity of multi-disciplinary teams of DR-TB experts. Also, conduction of enhanced cohort reviews helped to validate program data, identify performance gaps and best practices. The use of this experience to foster national PMDT expansion as well as the sustainability of these achievements is needed to further reduce the DR-TB burden in the country. However, due attention should be paid to the identified performance gaps for improvement. We propose this mixed model of care that applies a standardized minimum package of interventions in similar settings with similar challenges. This approach may have financial implications, so, the implied cost of the intervention may need to be investigated before the adoption. The financial analysis for the program was not considered for the scope of this study.

## Supporting information

S1 Table MDR-TB performance indicators: 2013–2017 against 2012 baseline. (DOCX)

S1 Dataset Program dataset MDR. (XLS)
