# Supplementary material for: Addressing the drug-resistant tuberculosis challenge through implementing a mixed model of care in Uganda
Source: PLoS One. 2020 Dec 29;15(12):e0244451. doi: 10.1371/journal.pone.0244451 (PMC7772013; doi:10.1371/journal.pone.0244451)
Supplement: S1 Table — (DOCX) [file pone.0244451.s001.docx]

**S1 Table. MDR-TB performance indicators: 2013 – 2017 against 2012 baseline.**

| **Year** | **2012** | **2013** | **2014** | **2015** | **2016** | **2017** | **Target** |
| --- | --- | --- | --- | --- | --- | --- | --- |
| (%) PMDT annual work plan developed and implemented | 0 | 70% | 100% | 100% | 100% | 100% | 75% |
| No. of HCWs seconded to DR-TB sites | 0 | 25 | 25 | 25 | 25 | 25 | 25 |
| No. of sites with Audiometry machines | 0 | 12 | 13 | 13 | 13 | 13 | 15 |
| No. of sites with a GeneXpert installed | 24 | 39 | 72 | 111 | 112 | 131 | 131 |
| National GeneXpert utilization (tests per machine) | unknown | 2 | 3 | 5 | 5 | 6 | 12 |
| No. of DR-TB sites using electronic registers | 0 | 12 | 15 | 15 | 15 | 15 | 15 |
| No. DR-TB sites using DR-TB MIS | 0 | 0 | 0 | 0 | 15 | 15 | 15 |
| No. of DR-TB sites conducting quarterly cohort reviews | 3 | 12 | 15 | 15 | 15 | 15 | 15 |
| No. of DR-TB sites remodeled/constructed | 1 | 2 | 3 | 4 | 6 | 6 | 5 |
| No. of DR-TB sites having a TB IC plan | 3 | 12 | 15 | 15 | 15 | 15 | 15 |
| No. of DR-TB sites supported with patient incentives/enablers | 3 | 15 | 15 | 15 | 15 | 15 | 15 |

PMDT - Programmatic Management of drug Resistant Tuberculosis, DR-TB - Drug-resistant tuberculosis, HCWs – Health Care Workers, MIS – Management Information System, TB – Tuberculosis, IC – Infection Control
